# Trade-off between soot and NO emissions during enclosed spray combustion of jet fuel

**DOI:** 10.1038/s41598-024-73484-8

**Published:** 2024-09-28

**Authors:** Una Trivanovic, Sotiris E. Pratsinis

**Affiliations:** Particle Technology Laboratory, Institute of Energy & Process Engineering, Department of Mechanical and Process Engineering, ETH Zürich, Sonneggstrasse 3, 8092 Zurich, Switzerland

**Keywords:** Fossil fuels, Aerospace engineering, Chemical engineering, Mechanical engineering

## Abstract

Aviation emissions of soot and nitrogen oxides are strictly regulated as they adversely impact human health and the environment. Jet fuel combustion conditions that decrease one pollutant concentration increase the other. Although it is not impossible to achieve both low soot and NO_x_ through clever design, it is hard to simultaneously reduce both. Although it is difficult to study such conditions due to high temperatures and gas flowrates of aircraft engines, recently it was shown that Enclosed Spray Combustion (ESC) of jet fuel results in soot with similar characteristics to that from aircrafts making ESC an attractive unit for studying aviation-like emissions. Furthermore, judicious swirl-injection of air downstream of the ESC burner drastically reduces soot emissions. Here the trade-off between NO and soot emissions during combustion of jet fuel is studied for the first time, to the best of our knowledge, accounting for the detailed structure of soot. Injecting air shortly after the ESC burner decreases soot but increases NO emissions, while such injection further downstream has the inverse outcome. This interplay between soot and NO emissions was correlated quantitatively with the gas temperature shortly after air injection. Consequently, combustion conditions for an optimal trade-off between soot and NO emissions for the ESC conditions studied here are identified that are at or below the lowest NOx emissions per unit mass of fuel from existing aircraft engines.

**Supplementary Information:**

The online version contains supplementary material available at 10.1038/s41598-024-73484-8.

## Introduction

Aviation is an important source of pollution including particulate matter and oxides of nitrogen (NO_x_) impacting air quality near airports^1^. Furthermore, such non-CO_2_ emissions have a significant impact on climate. They account for about two-thirds of aviation’s impact on net radiative forcing^2^. There is a large uncertainty associated with the non-CO_2_ RF^2^. In particular, the role of soot in contrail formation is still an active area of research^3^. So, the International Civil Aviation Organization (ICAO) has long regulated the emissions of NO_x_ and more recently of non-volatile particulate matter, nvPM^4^. The regulatory term nvPM refers to particles that remain solid when heated to 350 °C^4^. Particulate Matter (PM) from aviation can contain volatile particles such as sulfur and organics but nvPM is primarily soot^5^. Soot refers to carbonaceous nanoparticles that result from incomplete combustion of hydrocarbons. Reducing soot and NO_x_ emissions simultaneously is challenging as combustion conditions which reduce one typically promote the formation (or limit the decrease) of the other as has long been demonstrated in diesel engines^6^. Thus, the design of aircraft combustors which reduce both soot and NO_x_ while also maintaining strict safety and performance standards is challenging and trade-offs must be carefully weighed.

Jet fuel is sprayed into aircraft combustors creating locally fuel-rich conditions that result in incomplete combustion even if the global air-to-fuel ratio is fuel-lean. So, soot is formed largely by incomplete combustion in locally fuel rich regions at relatively low temperatures and ideally removed through oxidation at higher ones while NO_x_ formation is enhanced at high temperatures. The competition between agglomeration and oxidation determines the emitted soot particle size, morphology and concentration. In many combustion systems soot is formed but oxidized before its release^7^. Combustion systems can promote the oxidation of the surviving soot by increasing the flame temperature or O_2_ concentration which will in turn increase the soot oxidation rate^8^. The air-to-fuel ratio in a combustion system can be defined by its Effective eQuivalence Ratio (EQR) where EQR is the actual air-to-fuel ratio divided by the stoichiometric air-to-fuel ratio in the enclosure so, EQR = 1 denotes a stoichiometric flame and EQR > 1 a fuel-rich flame. Lean, premixed conditions could prevent the formation of soot in the first place, but this technology has only recently been pursued in aviation while many engines still use the RQL technology^9^. Today, aircraft combustors which feature very lean (EQR < 1) combustion, for example the Twin Annular Premixing Swirler (TAPS) combustors constitute a promising technology for minimizing soot and NO_x_ formation^10^.

However, the high temperatures that promote such soot oxidation also drive the formation of thermal NO_x_^11^. Laboratory flames are often used to study emissions from combustion systems and Enclosed Spray Combustion (ESC) of jet fuel has been shown to produce soot with similar properties (*d*_m_, *d*_p_, OC/TC) to that observed in soot from real aircraft engines^12^. The ESC is attractive compared to existing commercially available soot generators because, in addition to matching the properties of aircraft soot, it uses real liquid fuels rather than the gaseous fuels employed by most other generators. The ESC can provide important information necessary to fundamentally understand the mechanisms driving soot emissions from aircraft engines^13^ which still cannot be accurately captured by computational models of aircraft combustors^14^. Recently it was shown that swirl-injection of increasing concentrations of O_2_ into ESC of Jet A fuel can reduce soot total number concentration, *N*_tot_, and volume fraction, *f*_v_, by up to 95.4 and 99.6%, respectively^15^. This is similar to the concept of Rich-Quench-Lean (RQL) aircraft combustors which are comprised of an initial fuel-rich phase, followed by quenching with air to form a lean-burn zone^9^. The RQL concept was originally developed to lower NO_x_ emissions by rapidly reducing the temperature and prevent the formation of thermal NO_x_^16^ rather than to eliminate soot. If temperatures are dropped too low, soot cannot be efficiently oxidized leading to increased soot emissions^9^. So, the quenching stage in RQL combustors must be optimized to achieve temperatures that minimize both soot and NO_x_.

Several studies have used lab-scale RQL burners to investigate the effect of air dilution on soot concentrations^17^, mobility size distributions^14^, nanostructure, composition and morphology^15^. Similarly, models of NO_x_ formation in RQL combustors have been developed^18^. However, to the best of our knowledge there are no systematic studies which examine the effect of air quenching and its location in an RQL-like system on both the characteristics of soot, including the mobility, *d*_m_, and primary particle diameters, *d*_p_, as well as NO_x_ emissions from real jet fuels^19^. This is particularly important as failing to account for the real morphology of soot can lead to significant errors in calculations of the soot volume fraction^20^ and the radiative forcing of soot^21^. So, there is a lack of systematic laboratory studies on NO_x_ and soot emissions from real jet fuels which are necessary for fundamental understanding and modeling of emissions from real jet fuels. Here, the impact of air quenching location on the resulting soot and NO emissions during ESC of jet fuel is investigated while exploring optimal conditions to minimize emissions of both pollutants.

## Experimental

### Particle synthesis and sampling

**Fig. 1:**
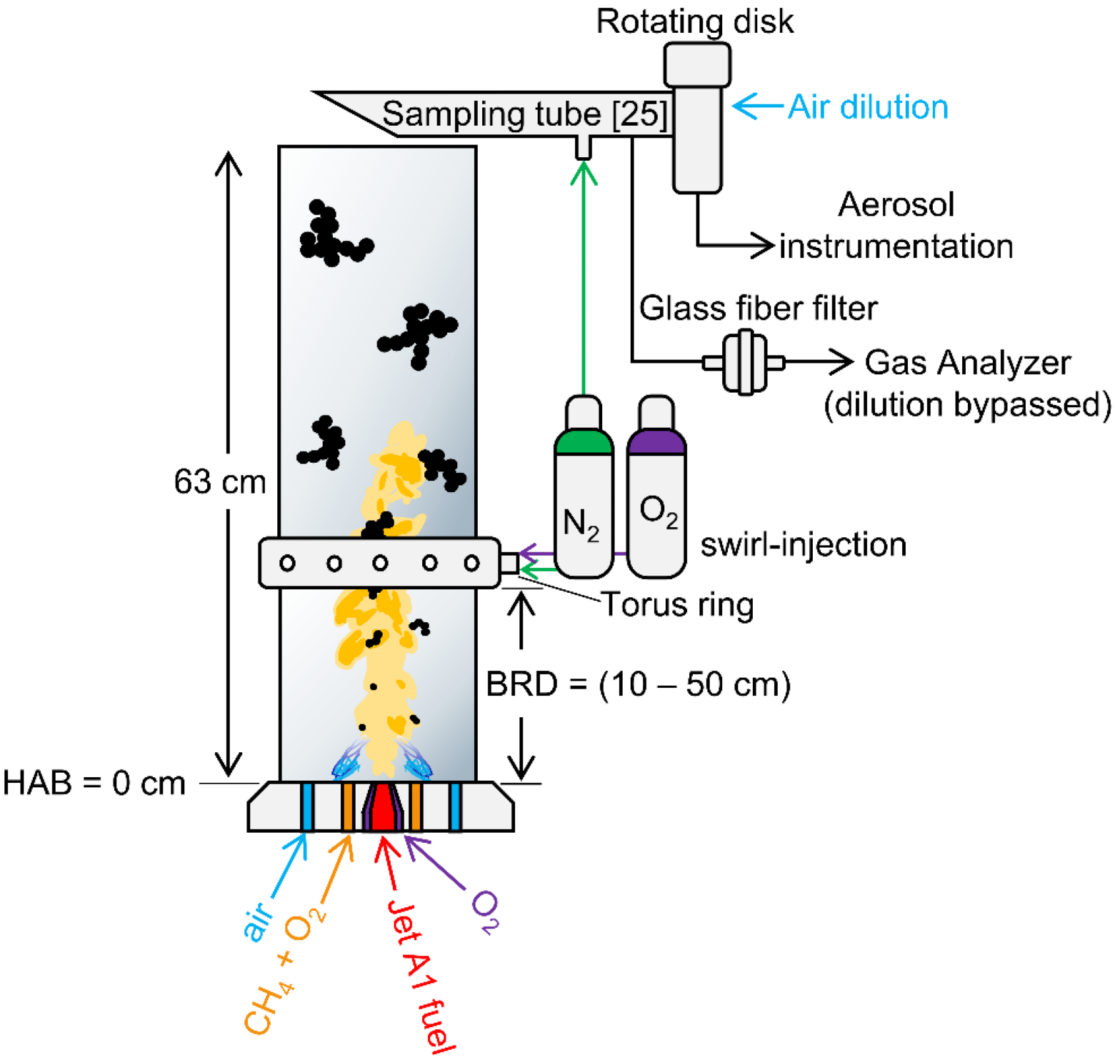
Schematic (not-to-scale) of the experimental set up. Jet A1 fuel from a capillary tube is dispersed by O_2_ into a fine spray that was ignited by a small, premixed methane flame. All of this was enclosed in two quartz glass tubes in series that are separated by a torus ring^24^ totaling 63 cm in length. The burner surface has a Height Above the Burner (HAB) = 0 and all other HAB are specified with respect to this location. The Burner to Ring Distance (BRD) is determined by the length of the first tube. The torus ring swirl-injects a mixture of N_2_ and O_2_ at 20 L/min to quench and dilute the flame. Exhaust is sampled immediately after the second quartz glass tube^25^ for online and offline analysis.

Soot and NO were generated by ESC of Jet A1 fuel (Birrfeld Airport, Lupfig, Switzerland)^12^, as depicted in Fig. 1. Here, 4 mL/min of fuel flowed through a capillary tube and 1 L/min of O_2_ from the concentric annulus to disperse the fuel into a fine spray. This is ignited with a small, premixed methane flame (CH_4_ = 1.25 L/min, O_2_ = 2.25 L/min) and surrounded by a 17 L/min sheath flow of air. This results in an EQR of approximately 1.78 accounting for all flows entering the system at a Height Above the Burner, HAB = 0, as described in detail elsewhere^12^. The premixed fuel (CH_4_)-lean flame serves to ignite and support the spray combustion^22^ but does not produce soot as its EQR is less than unity. Rather, the formation of soot is driven by the fuel spray droplets as their size affects the median *d*_m_, *d*_p_, and *N*_tot_ of soot^13^ (Fig. S2). Similarly, NO_x_ formation depends on flame temperature which in turn depends on the EQR^23^ that accounts for both methane and jet fuel [12; SI]. These conditions were chosen to match those in the literature^15^ which were shown to result in soot with similar morphology and OC/TC ratio to that observed in soot from aircraft engines while maintaining a sufficiently large mass concentration for studying the oxidation of these particles^15^. The flows at HAB = 0 and initial EQR are held constant throughout the experiments. Line loss calculations are presented in the SI.

The system was enclosed in two quartz glass tubes separated by a torus ring supplying a 20 L/min mixture of N_2_ and O_2_ in an upward, swirled motion^24^. When N_2_ only is fed to the ring, this quenches and dilutes the flame preventing soot oxidation^12^. Adding O_2_ to the ring allows for controlled oxidation of the soot in the second tube^15^. Here, 0 (N_2_ only), 5, 10, 15 and 20 vol% O_2_ (air) are used. Various vol% of O_2_ are studied to better understand how the O_2_ concentration affects soot and NO_x_ generation. However, the focus is on the addition of air as it is the most realistic to implement. All gases and jet fuel are supplied to the burner at room temperature (~ 20 ℃).

The Burner to Ring Distance (BRD) denotes the distance from the burner (HAB = 0 cm) to the bottom surface of the torus ring. The BRD was adjusted by using various tubes with lengths of 10, 20, 30, 40 and 50 cm to achieve a combined length of 63 cm when accounting for the two tubes plus the torus ring. All online measurements and offline samples are collected after the flame has been stabilized (at least 5 min after ignition) to ensure results are stable and condensation is avoided.

### Online measurements

The particle mobility size, *d*_m_, was measured with a Scanning Mobility Particle Spectrometer (SMPS) which consists of an X-ray neutralizer (TSI 3087), a Differential Mobility Analyzer (DMA, TSI 3081) and a Condensation Particle Counter (CPC, TSI 3775). To prevent coagulation, samples sent to the SMPS are diluted in the straight tube sampler first with a dilution factor of ~ 41.3 using nitrogen and air from a rotating disk diluter (MD 19-1E, Matter Engineering AG). The dilution was kept the same for all conditions to allow for a robust comparison between different conditions. The dilution flow cools the sampling probe to prevent particle thermophoretic, diffusion and coagulation losses as well as potential oxidation of the particles^25^. Full details of the straight tube sampler and the representativeness of the samples are given by Goudeli et al.^25^. The resulting mobility size distributions were fit to a log normal distribution to obtain the median mobility diameter, *d*_m_, Geometric Standard Deviation (GSD) and total number concentration, *N*_tot_, of the distribution. This way, the final results are not skewed by particles which were not measured due to the size limits of the SMPS.

Undiluted exhaust from the straight tube sampler was passed through a glass fiber filter to remove particles from the flow for gas analysis with an infrared photometer (ABB EL3040 with Uras26) sampled at a rate of 1 Hz. This allowed for measurement of Nitric Oxide (NO), in mg/m^3^ and converted to parts per million (ppm) assuming room temperature (25 °C) and pressure (1 atm).

The temperature of the flame, *T*, was measured with an R-type thermocouple (Intertechno-Firag AG) with a 1 mm (nominal) bead diameter and corrected for radiative losses^13^. The in-flame temperatures were measured by replacing the typical quartz glass tube with a steel tube containing sealable sampling ports every 5 cm. Although the thermocouple has not been shown to significantly influence the particles produced by the flame^13^, no other measurements were taken while the thermocouple was inserted in the flame.

### Offline characterization of soot

Particles deposited on glass fiber filters were used for offline analysis including Transmission Electron Microscopy (TEM, FEI Tecnai F30 FEG). Particles from the filter were dispersed in ethanol and then were placed in an ultrasonic bath to break up large agglomerates for 15 min^26^. After the ultrasonic bath, a drop of the ethanol solution was placed on a lacey carbon TEM grid with a 200 copper mesh support (LC200-Cu-150, Electron Microscopy Sciences). The resulting images were used to measure the primary particle diameter, *d*_p,_ of soot by manually placing ellipses over the primary particles with the software ImageJ^27^. From this, the area-equivalent diameter is calculated from more than 200 primary particles at each condition. At least 200 primary particles were counted as the median *d*_p_ leveled off by then as shown in Fig. S1 of the Supplementary Information (SI) and consistent with microscopy analysis of soot^28^ and TiO_2_^29^. Exemplary TEM images are shown in Fig. S2.

## Results and discussion

### Flame temperature

**Fig. 2:**
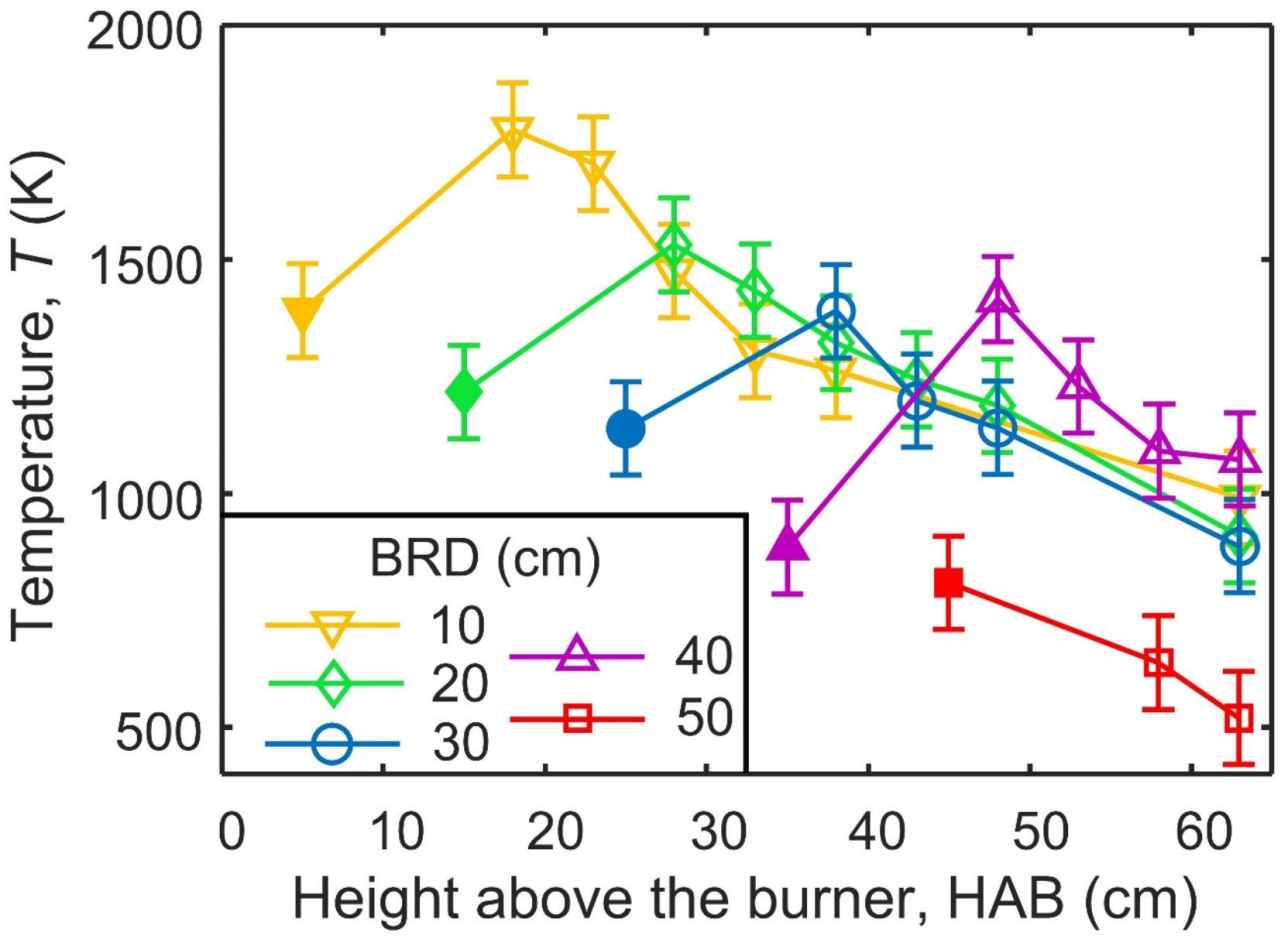
Centerline temperature by ESC of jet A1 fuel before (filled symbols) and after swirl-injection of air (open symbols) through a torus ring at BRD = 10 (inverse triangles), 20 (diamonds), 30 (circles), 40 (triangles) and 50 cm (squares).

Figure 2 shows the centerline flame temperatures 5 cm before (filled symbols) and after swirl-injection of air (open symbols) at BRD = 10 (inverse triangles), 20 (diamonds), 30 (circles), 40 (triangles) and 50 cm (squares). The temperatures at BRD = 30 cm (circles) are consistent with the corresponding literature^15^. All temperatures 5 cm before air injection, *T*_pre_, continuously decrease from 1350 K at BRD = 10 cm to 811 K at BRD = 50 cm, as all these locations are after the maximum temperature of ESC^13^: Fig. 1]. Immediately after air injection, the temperature increases at all BRD (except 50 cm) indicating the oxidation of unburned jet fuel and/or soot before dropping further downstream as expected. With BRD = 50 cm there is no such maximum in *T* as the temperature is too low already for further fuel/soot oxidation. In fact, this temperature profile is identical to that measured when injecting only N_2_ (Fig. S3a), proving that no fuel/soot oxidation takes place.

### Soot concentration and size

**Fig. 3:**
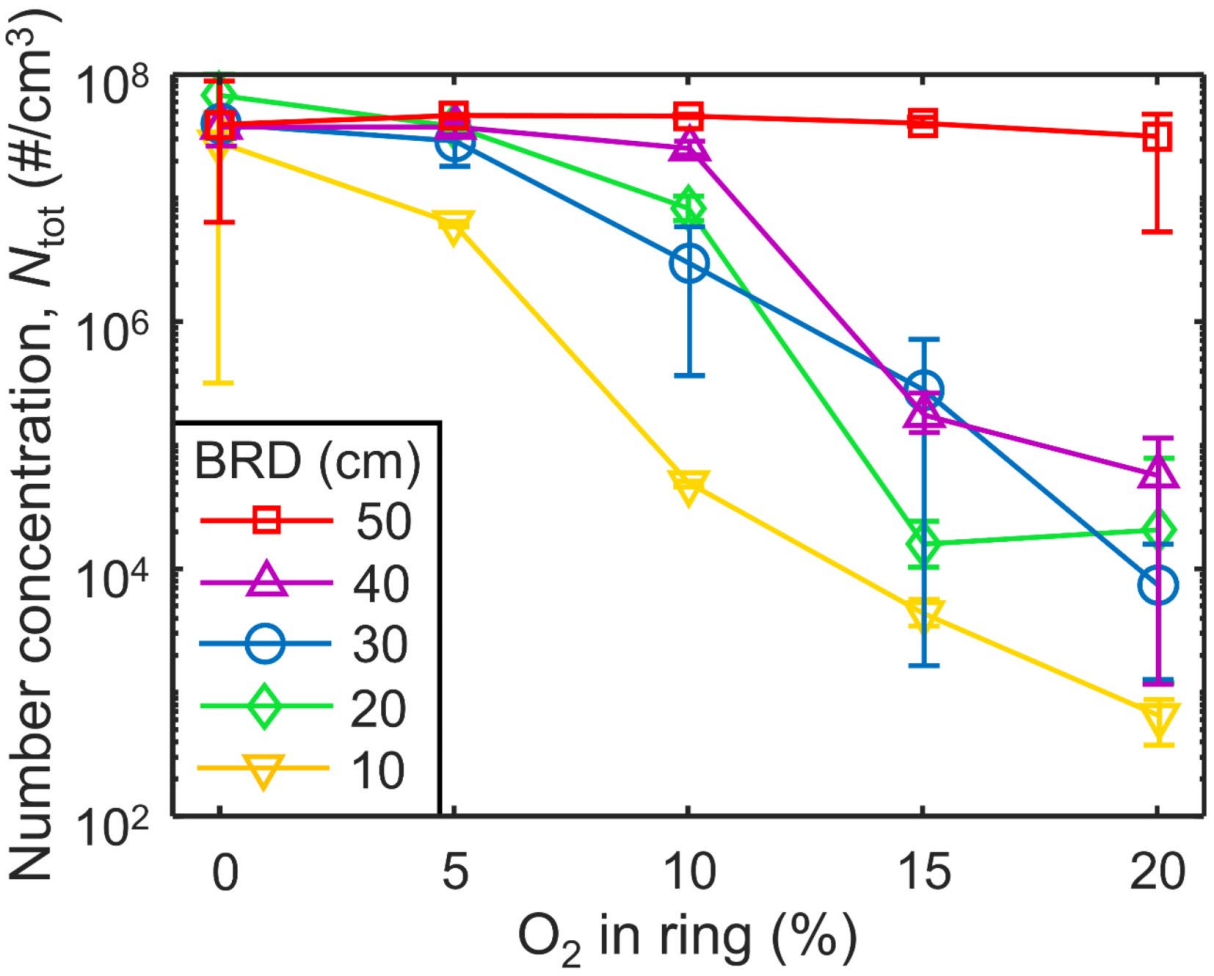
The total soot number concentration, *N*_tot_, corrected for the dilution factor and fit to a log normal distribution, as a function of the volume fraction of O_2_ supplied through the torus ring at BRD of 50 (squares), 40 (triangles), 30 (circles), 20 (diamonds) and 10 cm (inverse triangles).

Figure 3 shows the total number concentration corrected for the dilution ratio and fit to a log normal distribution, *N*_tot_, of soot at the end of the enclosure (HAB = 63 cm) when BRD = 50 (squares), 40 (triangles), 30 (circles), 20 (diamonds) and 10 cm (inverse triangles) with various O_2_ volume fractions in N_2_ supplied through the torus ring. Error bars represent the variation between 10 SMPS scans. When the concentrations are extremely low (< 10^4^ #/cm^3^), a small change in the total number of particles could have a large impact on the average value and the true error is likely larger than the variation between SMPS scans. Therefore, the low soot concentration at 15% O_2_ when BRD = 30 cm is most likely within such variation. Higher O_2_ vol% lead to larger reductions in *N*_tot_, consistent with the literature^15^. The initial concentration with the torus ring supplying N_2_ only (0 vol% O_2_), was nearly the same for all five BRD, about 4 × 10^7^ #/cm^3^. At BRD = 50 cm, there is virtually no change in soot number concentration even when adding air (20 vol% O_2_). This is another indication of no soot oxidation when BRD = 50 cm and consistent with Fig. 2. Conversely, for BRD ≤ 40 cm, reductions of at least 99.6% in the number concentration are achieved by gas injection with 15 vol % O_2_ through the torus ring. At lower BRD, less O_2_ is needed to achieve significant *N*_tot_ reductions. For example, injecting just 10% O_2_ at HAB = 10 cm resulted in a 99.9% reduction in *N*_tot_. This is consistent with a model Rich Quench Lean (RQL) combustor burning ethylene exhibiting also a decrease in soot number and volume concentrations when dilution air is injected earlier on in the flame^30^. The approximately four orders of magnitude reduction in *N*_tot_ when air is injected compared to N_2_ is similar to that achieved by passing soot through a lean-premixed flame^31^ suggesting that the swirl-injection of gas by the torus ring here results in turbulent conditions and intense mixing akin to a premixed flame. Leaner conditions (smaller EQR) resulted in smaller soot median *d*_m_ and *N*_tot_^12^ (Fig. S7). However, leaner conditions still resulted in orders of magnitude higher *N*_tot_. For example, at EQR = 1.29, *N*_tot_ = 4.02 × 10^7^ #/cm^3^ while the injection of air at BRD = 30 cm resulted in *N*_tot_ = 7.00 × 10^3^ #/cm^3^.

**Fig. 4:**
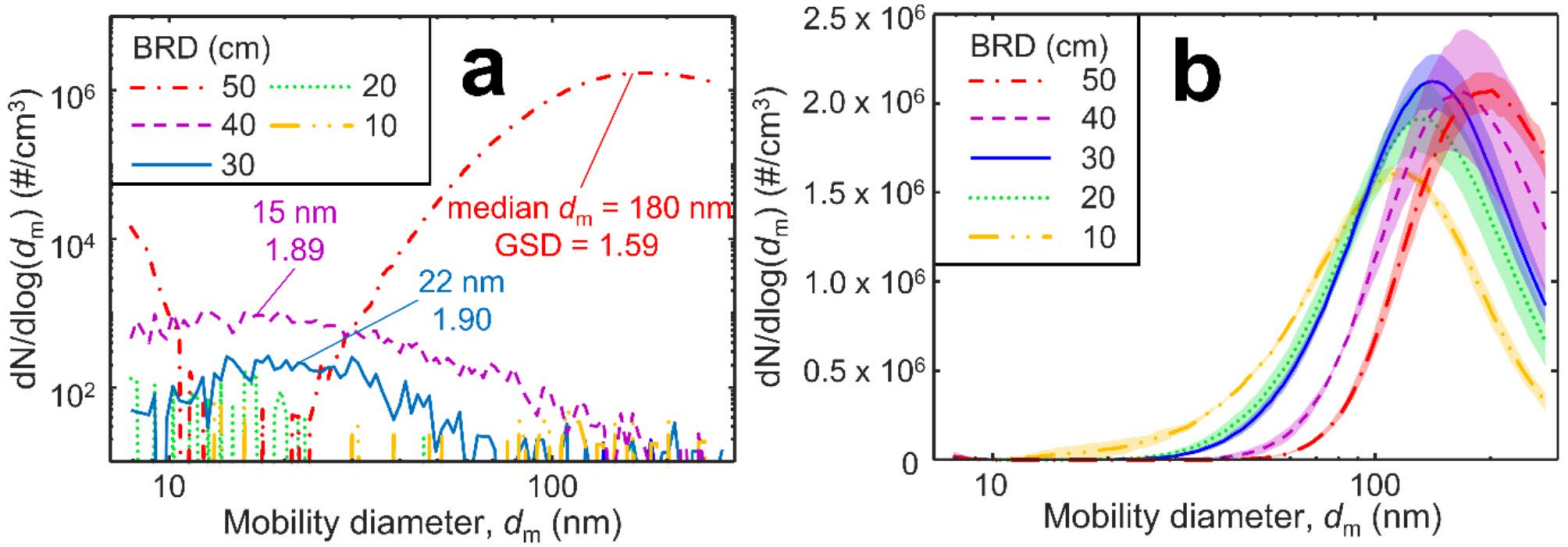
Exemplary soot mobility size distributions without correction for the dilution factor at BRD = 50 (dot-dashed line), 40 (dashed line), 30 (solid line), 20 (dotted line) and 10 cm (double dot-dashed line) with (a) air injection and (b) N_2_ injection. Shaded areas represent the variation between SMPS scans.

The soot mobility size distributions are shown in Fig. 4 BRD = 50 (dot-dashed line), 40 (dashed line), 30 (solid line), 20 (dotted line) and 10 cm (double dot-dashed line) when (a) air is injected and (b) N_2_ is injected. The shaded areas in Fig. 4b represent the variation between SMPS scans of the same condition. Prior to air injection (Fig. 4b), the median *d*_m_ = 196 ± 2.9, 170 ± 8.8, 142 ± 4.1, 133 ± 6.3 and 111 ± 2.9 nm at BRD = 50, 40, 30, 20 and 10 cm, respectively. The geometric standard deviation, GSD at these same BRD are 1.57 ± 0.01, 1.61 ± 0.01, 1.65 ± 0.01, 133 ± 0.01 and 1.71 ± 0.03. At BRD = 50 cm with air injection, the median *d*_m_ of 180 ± 1.2 nm is close to that obtained when pure N_2_ is fed through the torus ring. It is possible that the smaller peak below 10 nm when BRD = 50 cm is an artifact due to large particles left in the SMPS which are then counted as small particles on the subsequent scan. The GSD of 1.59 ± 0.01 is slightly above the quasi-self-preserving GSD = 1.48 ± 0.03 for flame reactors^32^. In contrast, at BRD = 40 and 30, the distributions drop to median *d*_m_ = 15 ± 2.7 and 22 ± 2.9 nm, respectively, and the concentration is significantly reduced for more than 10 vol % O_2_ in the swirl gas. This drastic reduction in size and concentration suggests that significant oxidation has taken place removing most of the soot originally produced and consistent with Fig. 3. The distributions have also widened to 1.89 ± 0.41 and 1.90 ± 0.20 at BRD = 40 and 30 cm, respectively. This widening could be also due to some fragmentation during soot oxidation. A similar widening of the size distribution has been observed from ESC of Jet A fuel (rather than A1)^15^ during oxidation of soot from a premixed flame burning a JP-8 jet fuel surrogate^31^ and during simulations of diesel soot oxidation^33^. Further lowering the location of the torus ring to BRD = 20–10 cm, results in such low soot concentrations (*N*_tot_ < 50 #/cm after dilution) that a lognormal distribution cannot be fit to the results. The very low concentrations highlight how effective the injection of air is at removing soot particles from the system when BRD ≤ 40 cm.

Evidently, injection of N_2_ containing even small amounts of O_2_ when BRD = 10 cm is most effective for removing soot. The high temperatures there (Fig. 2, inverse triangles) increase the soot oxidation rate^34^ but other factors may also contribute to the fast removal of soot. A BRD = 10 cm coincides with the maximum temperatures measured in these flames at HAB ~ 10 cm (Fig. S3a-d) ranging from 1391 to 1878 K. Soot inception in laminar diffusion flames begins in a similar temperature range of 1332–1913 K for various hydrocarbons^11^. Qualitatively, soot begins to deposit visibly on the walls of the quartz glass tubes near HAB = 10 cm (Fig. S4). In addition, while the *N*_tot_ from injection of pure N_2_ (0 vol% O_2_ injected) was practically identical for all BRD (Fig. 3), the initial mobility size distributions, Fig. 4b, shifted to smaller mobility diameters when pure N_2_ was injected at lower BRD (i.e. earlier in the flame). This N_2_ essentially diluted the soot aerosol and reduced its coagulation-agglomeration rate. The primary particle sizes with such N_2_ quenching were similar for all BRD as well, Fig. S5a, suggesting that agglomerates formed at lower BRD contained fewer primary particles resulting in a lower volume fraction (Fig. S6, open symbols). This suggests that quenching at BRD = 10 cm interferes with soot formation. Furthermore, less mature soot particles tend to have more disordered carbon^35^ which is easier to oxidize compared to ordered material^36^. When BRD ≥ 20 cm, the *T*_pre_ ≤ 1217 K so injecting air quite likely does not interfere with soot formation.

### Nitric oxide emissions

**Fig. 5:**
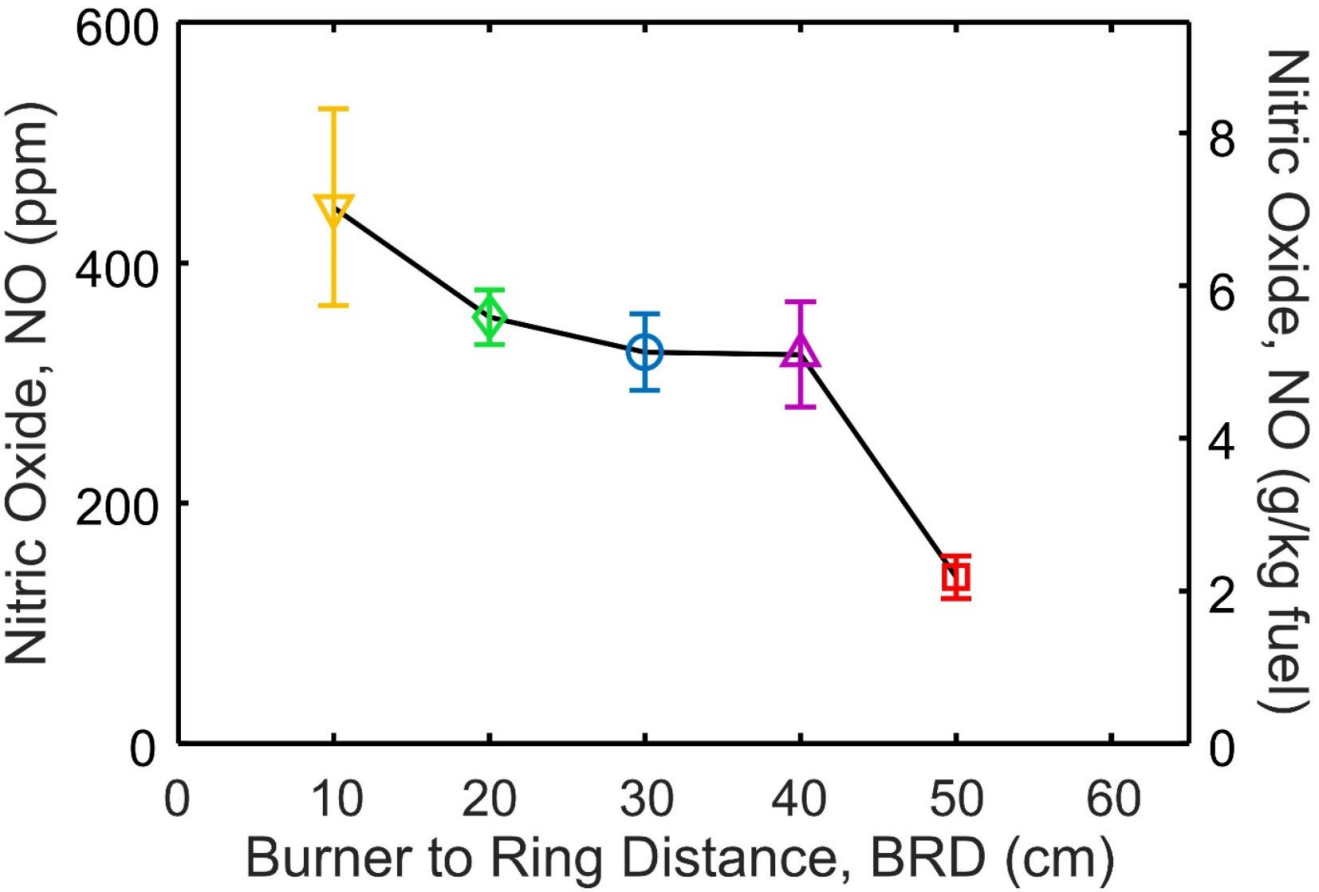
The nitric oxide (NO) emissions in parts per million (left axis) or normalized by the amount of fuel that would be burned over the 32.9 min landing and take-off (LTO) cycle (right axis) as a function of the BRD of swirl-injection of air.

Figure 5 shows the nitric oxide, NO, emissions at the end of the ESC enclosure when BRD = 10 (inverse triangles), 20 (diamonds), 30 (circles), 40 (triangles) and 50 cm (squares). The error bars represent the standard deviation between at least three experiments. There is a nearly linear trend between BRD and NO with the highest NO emissions of 446 ± 82 ppm occurring at BRD = 10 cm and the lowest NO = 138 ± 18 ppm at BRD = 50 cm. In this regard, injecting air later in the flame is desirable to minimize NO. Increasing the O_2_ concentration from 0 to 20% results in also a linear increase in NO from 208 ± 35 ppm with pure N_2_ quenching up to 325 ± 32 ppm with air quenching when BRD = 30 cm (Fig. S7). This is expected as lower BRD increased post-injection temperatures (Fig. 2) while N_2_ quenching reduced them (Fig. S3) and increased NO has long been associated with higher temperatures due to the Zeldovich mechanism of NO formation^37^.

The NO_x_ emissions from aviation are typically expressed as grams of NO_x_ produced over the standardized Landing and Take-Off (LTO) cycle normalized by the kilograms of fuel burned over this time (g NO_x_/kg fuel)^4^. While the laboratory burner cannot be cycled through the LTO cycle, some comparison to real values is necessary to put the quantities measured here in context with the literature. Therefore, to provide some context for these results we convert the NO emissions measured here to g NO/kg fuel for the same length of time as the LTO cycle (32.9 min) resulting in values of 7.03 ± 1.29, 5.59 ± 0.036, 5.13 ± 0.50, 5.10 ± 0.69 and 2.18 ± 0.28 g/kg when BRD = 10, 20, 30, 40 and 50 cm, respectively (Fig. 5, right axis). The permissible NO_x_ from an engine depends on both its maximum rated thrust and pressure ratio where more NO_x_ is permitted from engines with higher thrust and pressure ratios^4^. This allows for a balance between reducing fuel consumption through the use of more efficient engines (i.e. higher pressure ratios) and the air quality impacts of NO_x_^38^. The dependence on pressure ratio and rated thrust makes it difficult to compare the results here directly to regulatory limits. However, the present values are at the bottom or below the range of NO_x_ reported in the publicly available ICAO aircraft emissions databank for existing engines from approximately 5.20 to 279 g/kg over the LTO cycle^39^. It is important to note that the present system is orders of magnitude smaller than a real aircraft engine and the NO_x_ emissions of aircrafts tend to decrease with lower fuel flow in real engines^40^. Here, the fuel flow is 4 mL/min or 5.38 × 10^−5^ kg/s while the lowest fuel flow in the ICAO database is 0.023 kg/s^39^. Although higher fuel flowrates are associated with higher NO_x_ emissions from aircrafts, the magnitude of this effect seems to vary significantly between different engine types indicating that the NO_x_ can be reduced through engine design^40^. Thus, the relative magnitude and trend of NO emissions shown in Fig. 5 and S8 are more important than the absolute values as the temperatures, pressures and EQR here are not necessarily the same as those in real aircraft combustors.

**Fig. 6:**
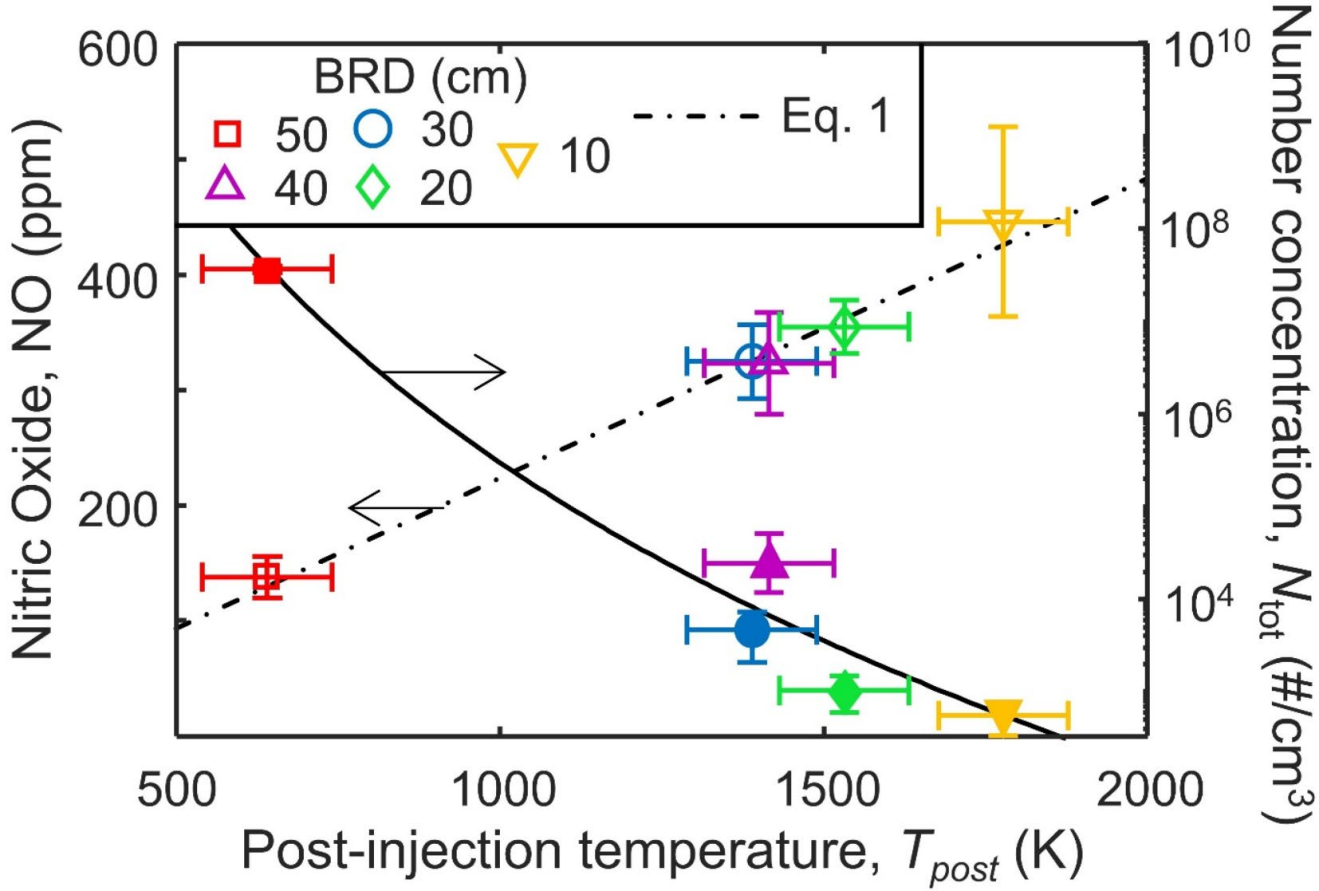
The temperature 5 cm after swirl-injection of air, *T*_post_, (Fig. 2), and the associated NO (open symbols) and total number concentration *N*_tot_, (filled symbols), corrected for the dilution factor and fit to a log normal distribution, emitted from the enclosure when BRD = 50 (squares), 40 (triangles), 30 (circles), 20 (diamonds) and 10 cm (inverse triangles).

Some NO is produced in the flame before the injection of air due to the first temperature peak at approximately HAB = 10 cm (Fig. S3) and presence of N_2_ in the sheath air. This is why some NO is still detected when BRD = 50 cm or when N_2_ is injected (Fig. S8). The so-called penalty for oxidizing soot is the NO formed during the second temperature peak post-air injection which adds to the NO formed earlier in the flame. For this reason, the NO emissions measured at the end of the enclosure are correlated to the temperature 5 cm downstream of the air injection as shown in Fig. 6 (left axis). When BRD = 50 cm (square), the post-injection temperature, *T*_post_, is the lowest, 640 K, and correspondingly the NO is also at its lowest, 138 ppm. For BRD = 20–40 cm, *T*_post_ = 1531 –1415 K which is within the standard deviation between measurements of approximately 100 K. Accordingly, the mean NO values measured vary from 355 to 324 ppm across the same range again with significant overlap in their standard deviations. At BRD = 10 cm, the post-injection temperature was significantly higher, 1777 K, and the emitted NO was 446 ppm. A linear relationship between NO and *T*_post_ can be described as:

1 $$\left[NO\right]=0.26 \cdot T_{post}-36$$

where [NO] is in ppm and *T*_post_ in K with an R^2^ = 0.99. So, NO increases at a rate of 0.26 ppm/K across the range studied here. This correlation holds true also when pure N_2_ is injected in the flame (Fig. S11) further confirming that the post-injection temperature is an important metric for determining the NO emitted rather than the maximum temperature which is similar for all BRD (Fig. S3).

The temperature after swirl-injection of air is inversely related to the concentration of soot produced (Fig. 6; filled symbols). When oxidation of soot occurs at high temperatures, the process is limited by the reaction rate and occurs primarily on the soot surface, shrinking the soot particles^41^. In contrast, at low temperatures the reaction occurs slowly enough for O_2_ to diffuse into the particle and cause internal oxidation which reduces the mass and increases the porosity of soot without a significant change in its structure and primary particle diameter^41^. The significance of internal oxidation starts around 973^42^ to 1073 K^34^. When BRD ≤ 40 cm, all *T*_post_ are significantly above this threshold.

When BRD = 50 cm, the temperature just before air injection is 811 K and drops well below the threshold for surface oxidation after the torus ring. This suggests that air injection at BRD = 50 cm does not induce oxidation and if it does, it should be internal oxidation preserving the size of the particles. Here, the primary particle diameter of soot produced when BRD = 50 cm is not affected by the composition of the quenching gas (Fig. S5b). The median *d*_m_ is only slightly decreased from 196 nm with N_2_ injection (Fig. 4b) to 180 nm with air (Fig. 4; dot-broken line). Also, with BRD = 50 cm only 10 cm are left to the end of the enclosure so, soot has less time to oxidize. On the other hand, at BRD = 30 cm the *d*_m_ is reduced significantly from 142 nm (Fig. 4b) with pure N_2_ injection down to just 15 nm with air (Fig. 4, solid line), a 90% reduction.

**Fig. 7:**
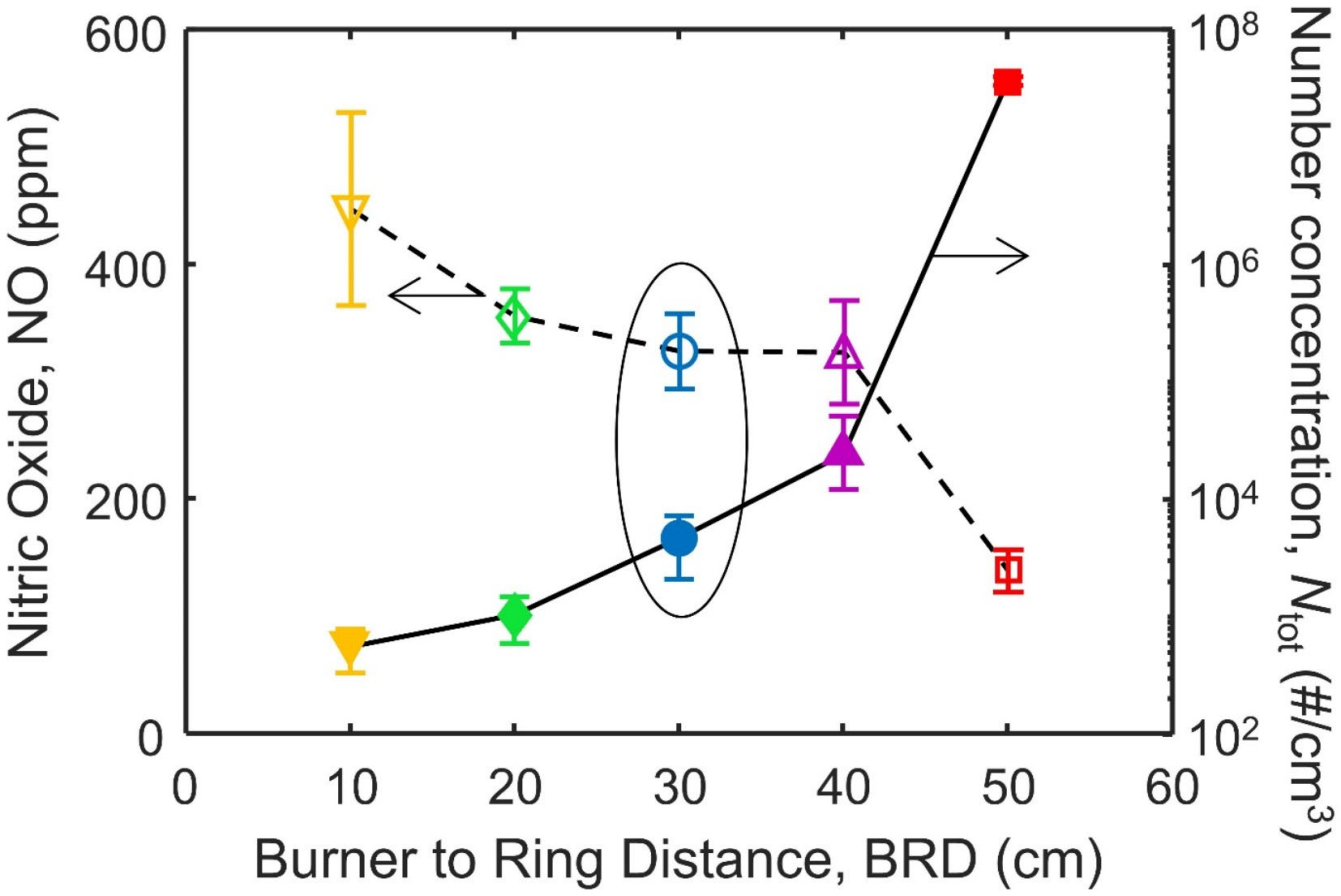
The NO (left axis, open symbols) and *N*_tot_ (right axis, filled symbols), corrected for the dilution factor and fit to a log normal distribution, measured at the exit of the enclosure when BRD = 10 (inverse triangles), 20 (diamonds), 30 (circles), 40 (triangles) and 50 cm (squares).

As both NO and soot are pollutants which must be minimized due to increasingly strict regulations, an optimal trade-off is needed. Figure 7 shows the NO (left axis, open symbols) and *N*_tot_ (right axis, filled symbols) as a function of BRD. The best trade-off between NO and *N*_tot_ is when BRD = 30 cm. The mean NO is nearly identical to that produced when BRD = 40 cm, however the *N*_tot_ of soot is lower. However, the NO and *N*_tot_ at BRD = 30 cm are similar to those achieved at BRD = 20 and 40 cm. When BRD is below 20 cm or above 40 cm, the trade-offs between soot and NO become more apparent and a significant penalty is paid either in increased NO (BRD = 10 cm) or increased soot (BRD = 50 cm). Interestingly, there is an abrupt change from significant soot oxidation and elevated NO at BRD = 40 cm to negligible oxidation and low NO at BRD = 50 cm.

The temperature just before the torus ring is similar at 885 and 881 K at BRD = 40 and 50 cm, respectively. After air injection, this changes significantly with *T*_post_ = 1415 and 640 at BRD = 40 and 50 cm, respectively. Thermal Gravimetric Analysis (TGA) of aircraft soot in air showed the onset of soot mass loss at around 700 K, which then dropped off steeply around 830 K^43^, similar to the temperature threshold observed here. In addition, TGA allows for a soot oxidation experiment where temperatures are ramped up slowly in a closely controlled environment while during ESC, the oxidation of soot releases heat which accelerates the oxidation further causing the increase in *T*_post_ relative to *T*_pre_ and significant differences in both *N*_tot_ and NO observed here. The exact temperature at which this tipping point is reached will depend in part on the structure of the particles^44^ which here has been shown to be similar to that of aircraft soot^13^. Thus, the location of air quenching can significantly impact both NO and soot emissions as both emissions are related to the temperature in the flame.

## Summary & conclusions

A torus ring was used to swirl-inject air into ESC of Jet A1 fuel at burner to ring distances, BRD = 10, 20, 30, 40 and 50 cm. Maximum flame temperatures occur at approximately 10 cm height above the burner so, when BRD = 10 cm, the highest temperature is 1777 K downstream of the torus ring. This BRD in turn results in the lowest soot concentrations, *N*_tot_ = 560 #/cm^3^ but the highest NO (446 ppm). At the other extreme (when BRD = 50 cm), post-injection temperatures are significantly lower, i.e. 640 K. Then, NO is significantly lower at 138 ppm, but the soot concentration is orders of magnitude higher at 3.7 × 10^7^ #/cm^3^. The temperatures, NO and *N*_tot_ measured with air quenching at BRD = 50 cm were nearly identical to that measured with N_2_ quenching. This suggests that negligible soot oxidation is taking place then. The post-injection temperature was the most important metric for determining NO emissions with a linear correlation for the range of temperatures studied here. At BRD = 20–40 cm, temperatures and thus NO emissions are quite similar with nearly identical means of 325 and 324 ppm obtained at BRD = 30 and 40 cm, respectively. The *N*_tot_ was reduced by more than four orders of magnitude for all three BRD, but the greatest reduction of six orders of magnitude was achieved at BRD = 20 cm resulting in only about 500 #/cm^3^. Thus, BRD = 30 cm offers the best trade-off between NO and soot emissions. Beyond it, temperatures are either too high, promoting NO formation or too low to oxidize soot. These results indicate the potential of optimal aircraft engine design and operation with respect to minimizing both NO and soot emissions through optimizing the aerosol residence time at high temperatures. This laboratory burner provides important information on soot morphology, concentration and NO emissions which are necessary for fundamentally understanding and modeling the emissions of jet fuel combustion.

### Supporting information

Variation of the median *d*_p_ by the number of particles counted; exemplary TEM images, expanded temperature profiles for all BRD with air and pure N_2_ injection; photo of the experimental set up, primary particle size distributions; volume fraction of soot; NO emissions at BRD = 30 as a function of the O_2_ vol % injected through the torus ring; NO concentrations with air and pure N_2_ injection as a function of BRD; NO concentrations with air and pure N_2_ injection as a function of *T*_post;_ line loss calculations.

## Electronic supplementary material

Below is the link to the electronic supplementary material.

Supplementary Material 1

## Data Availability

Data will be made available upon request of the corresponding author.
