# Supplementary material for: Trade-off between soot and NO emissions during enclosed spray combustion of jet fuel
Source: Sci Rep. 2024 Sep 28;14:22417. doi: 10.1038/s41598-024-73484-8 (PMC11438904; doi:10.1038/s41598-024-73484-8)
Supplement: Supplementary file 1 — Supplementary Material 1 [file 41598_2024_73484_MOESM1_ESM.docx]

Supporting information for

**Trade-off between soot and NO emissions during enclosed spray combustion of jet fuel**

*by*

*Una Trivanovic, and Sotiris E. Pratsinis**

Particle Technology Laboratory, Institute of Energy & Process Engineering,

Department of Mechanical and Process Engineering,

ETH Zürich, Sonneggstrasse 3, CH-8092 Zürich, Switzerland.

Ph. +41 (0) 44 632 31 8110; Fax. +41 (0) 44 632 15 95

*Corresponding author: Tel. +41 44 632 31 80 sotiris.pratsinis@ptl.mavt.ethz.ch

Figure S1 shows the comparison between the number of soot primary particles counted and the median primary particle diameter obtained from Transmission Electron Microscopy (TEM) for Burner to Ring Distances, BRD = 50 (red line), 40 (purple line), 30 (blue line), 20 (green line) and 10 (yellow line) cm. In each case, an asymptote is approached by 200 particles counted as has been observed previously for soot [29] and TiO_2_ [30]. Thus, more than 200 primary particles were sized for each condition.


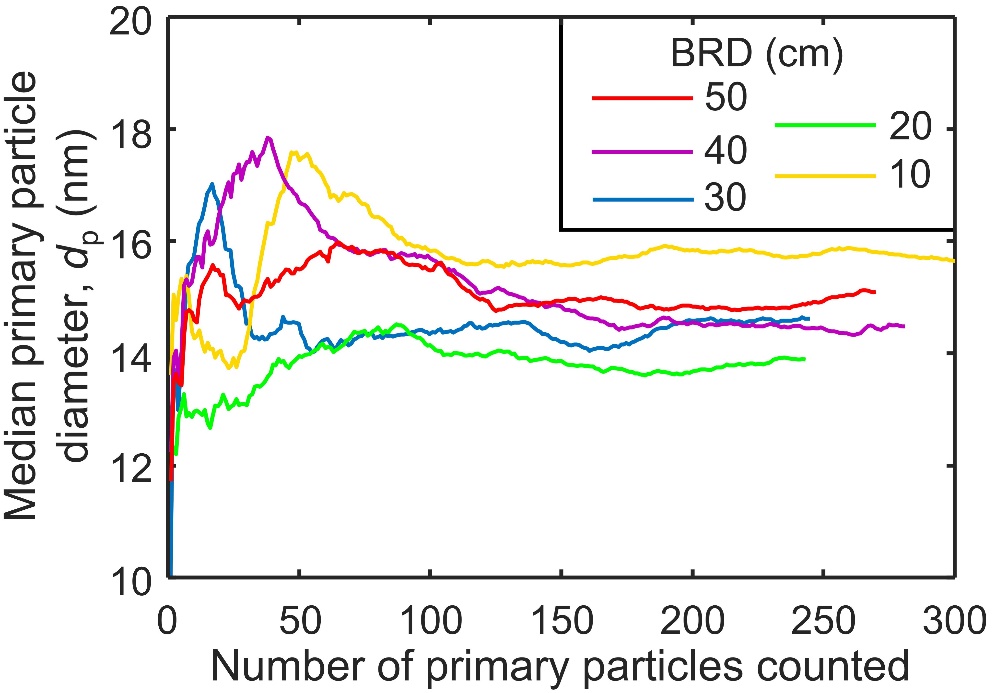


Figure S1: The median primary particle diameter, d_p_, as a function of the number of primary particles counted. Variation in the median levels off when approximately 200 particles were counted.

Example images used to obtain the results in Figure S1 are shown in Figure S2 for soot produced with N_2_ quenching at BRD = a) 10, b) 20, c) 30, d) 40 and e) 50 cm. Figure S2f shows soot produced with air quenching at BRD = 50 cm.


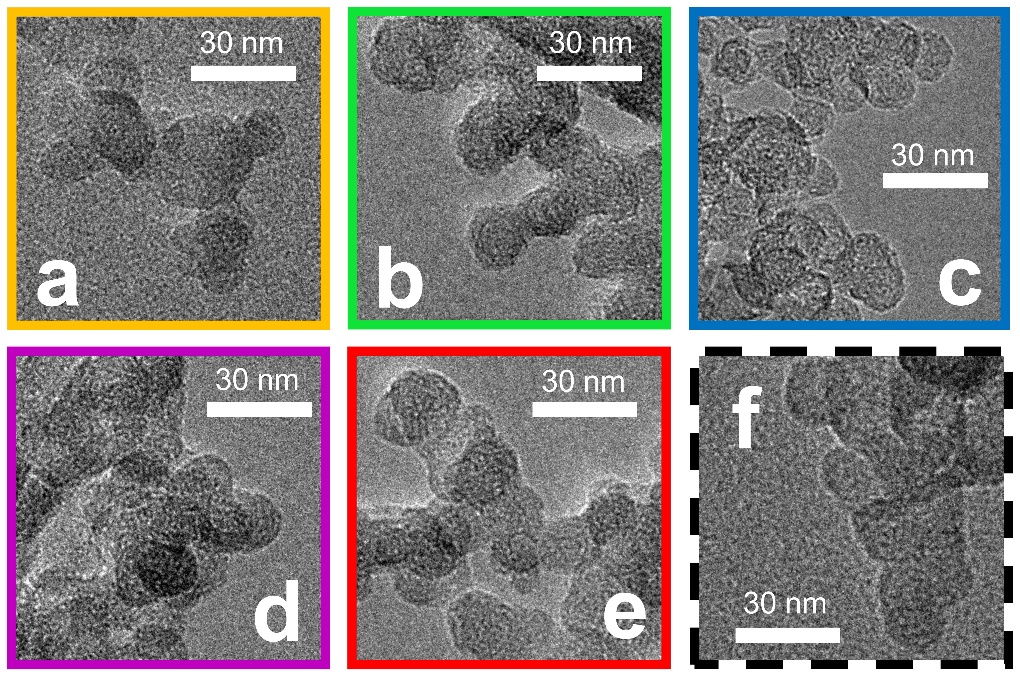


Figure S2: Exemplary TEM images of soot produced with pure N_2_ quenching at a) BRD = 10, b) 20, c) 30, d) 40 and e) 50 cm. The particles in f) were produced with air quenching at BRD = 50 cm.

Figure S3 shows the centerline temperature profiles for Enclosed Spray Combustion (ESC) of jet A1 fuel with swirl-injection of air (open symbols) and pure N_2_ (filled symbols) at BRD of a) 50 b) 40, c) 30, d) 20 and e) 10 cm.


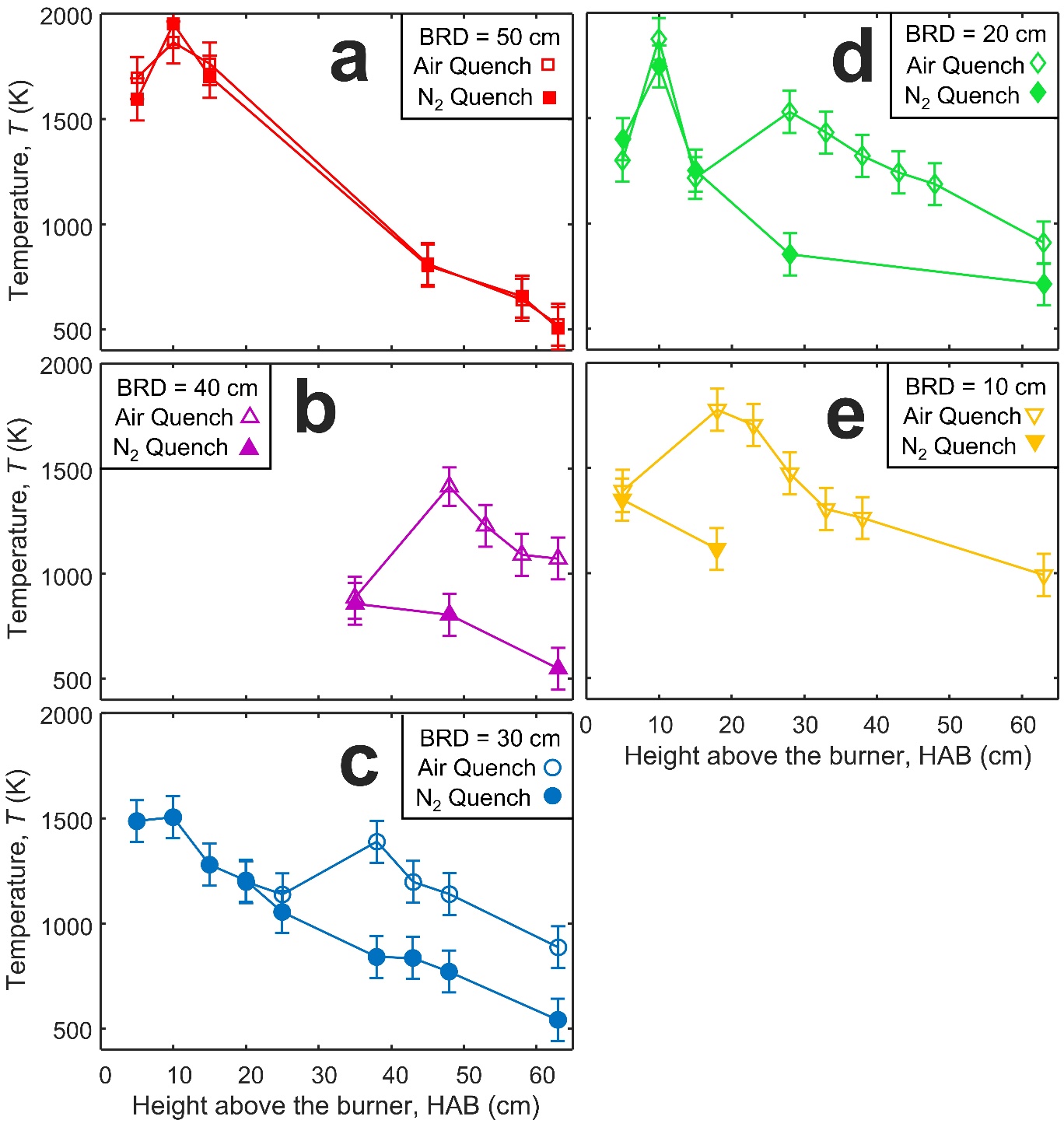


Figure S3: The centerline flame temperature from ESC of jet A1 fuel when pure N_2_ (filled symbols) or air (open symbols) are swirl-injected through a torus ring at BRD = a) 50, b) 40, c) 30, d) 20 and e) 10 cm.

A photo of the experimental set up is shown in Figure S5 with a BRD = 20 cm. The quartz glass tubes are clear but around HAB = 10 cm, soot begins to visibly accumulate on the walls of the enclosure. Air is injected through the torus ring oxidizing any remaining fuel and the soot produced earlier in the flame and therefore no soot is visibly accumulating on the enclosure walls downstream of the torus ring.


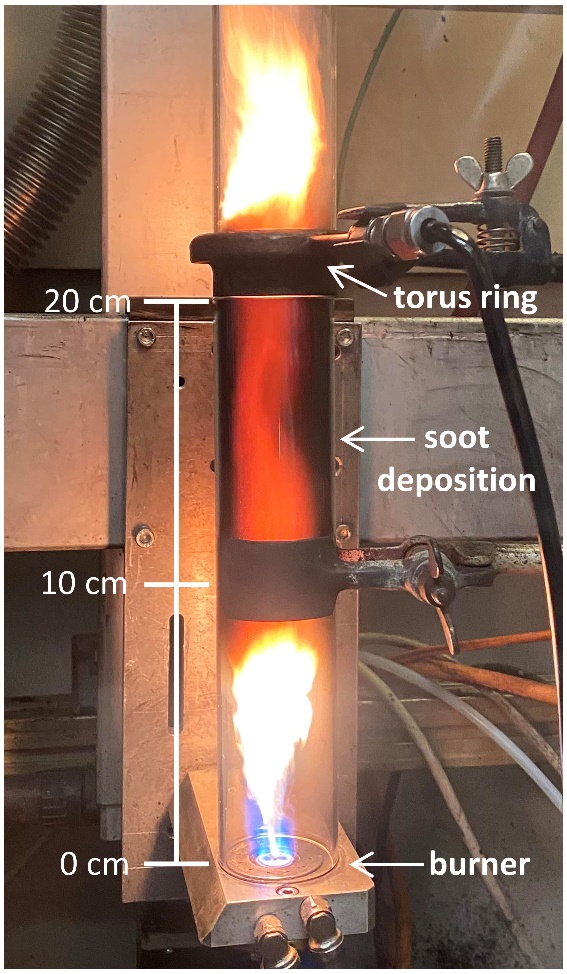


Figure S4: The experimental set up for ESC of jet A1 fuel with BRD = 20 cm and swirl injection of air. In the lower tube soot is formed and visibly deposits on the sides of the quartz glass tube. In the second tube the soot is oxidized and the tube is visually clean.

The primary particle size distributions of soot produced with 0% O_2_ injected through the ring (N_2_ only) are shown in Figure S6a at BRD = 50 (squares), 40 (triangles), 30 (circles), 20 (diamonds) and 10 cm (inverse triangles). There are no significant differences in primary particle size between soot produced at all BRD. For BRD ≤ 40 cm, the primary particle size after air injection could not be measured due to the extremely low concentrations. At BRD = 50 cm, a sufficient sample size of soot after air injection was obtained (Figure S6b, open squares). At this BRD, there was no significant difference between the primary particle sizes after pure N_2_ or air injection.


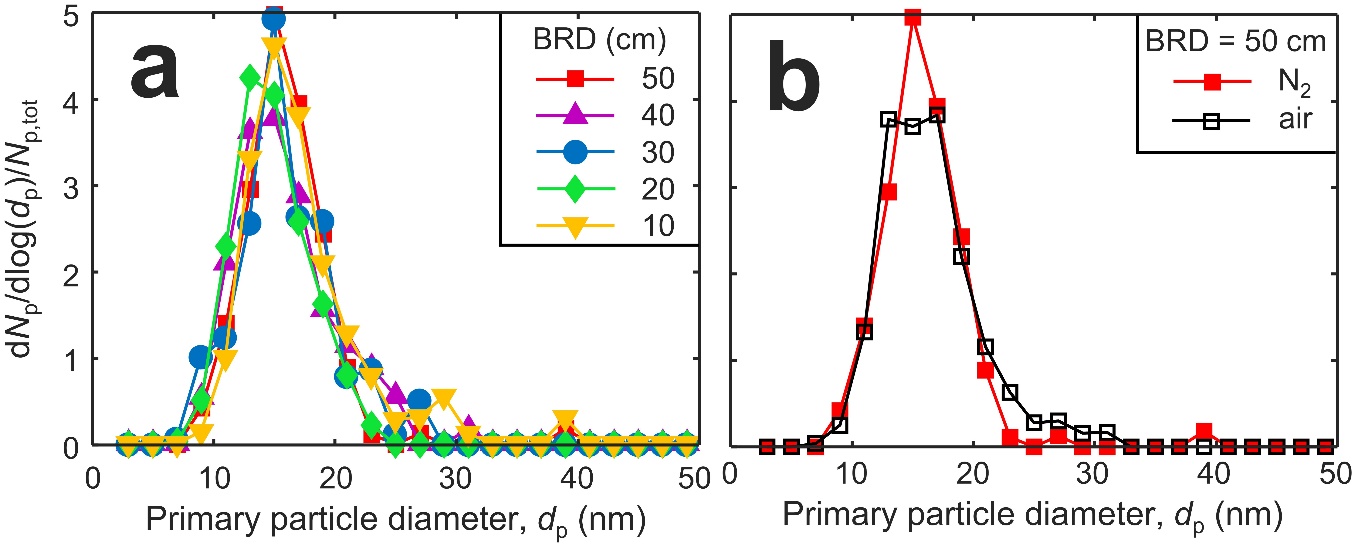


Figure S5: The primary particle size distributions obtained from TEM imaging with a) pure N_2_ quench at BRD = 50 (squares), 40 (triangles), 30 (circles), 20 (diamonds) and 10 cm (inverse triangles) having median primary particle diameters of 15.1, 15.2, 14.6, 13.8 and 15.7 nm, respectively and b) at BRD = 50 cm with pure N_2_ (red filled squares) and air (black open squares) which showed virtually no change compared to N_2_ quenching with a median primary particle diameter of 14.9 nm.

The volume fraction, *f*_v_, was estimated using the SMPS size distributions and primary particle sizes accounting for its agglomerated structure by [31]:

(Eq. S1)

$$f_{v}=\frac{\pi}{6}\sum_{i=1}^{k} N_{i}d_{m,i}^{2.22}d_{p}^{-0.78}$$

where *N*_i_ is the number concentration of soot with *d*_m,i_ and mean *d*_p_. The index k denotes the *d*_m_ bins measured with the SMPS and varies from 1 to 100. At BRD ≤ 40 cm with air quenching, soot quantities were too small to obtain a sufficient sample for analysis. So, the *d*_p_ was estimated by assuming that it was reduced by 17%, when compared to the *d*_p_ produced at the same BRD with N_2_ quenching [15]. Figure S7a shows both the pure N_2_ (filled symbols) and estimated air *f*_v_ (open symbols). At BRD = 50 cm, there is significant overlap which makes it impossible to distinguish between symbols for pure N_2_ and air injection. Figure S7b zooms in on just the *f*_v_ after pure N_2_ injection which does show a slight increase with BRD. This is because the mobility size distributions shifted to slightly larger sizes at larger BRD although the total number concentration stayed approximately constant at 4 x 10^7^ #/cm^3^ (Figure 3).


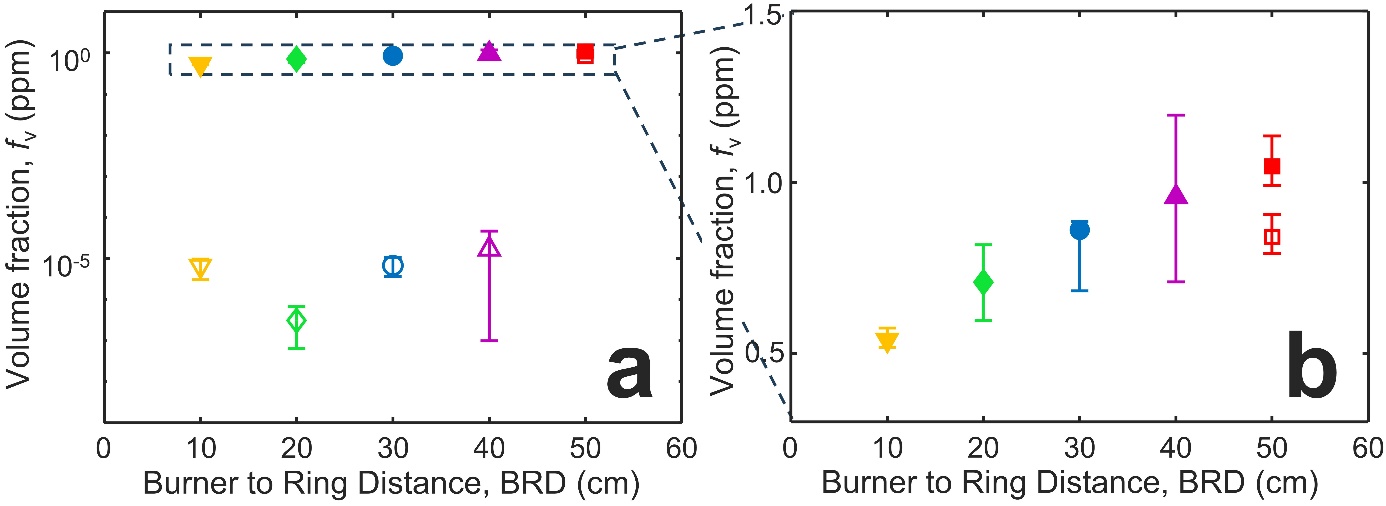


Figure S6: The estimated emitted volume fraction, f_v_, of soot a) with air (open symbols) and pure N_2_ (filled symbols) and b) a linear scale for the latter as a function of the BRD.

The NO emissions produced with various O_2_ volume fractions injected through the torus ring at BRD = 30 cm from 0 to 20 vol% O_2_ are shown in Figure S8. There is a steady increase in NO as the O_2_ fraction increases as would be expected based on the simultaneously decreasing soot number concentration (Figure 3).


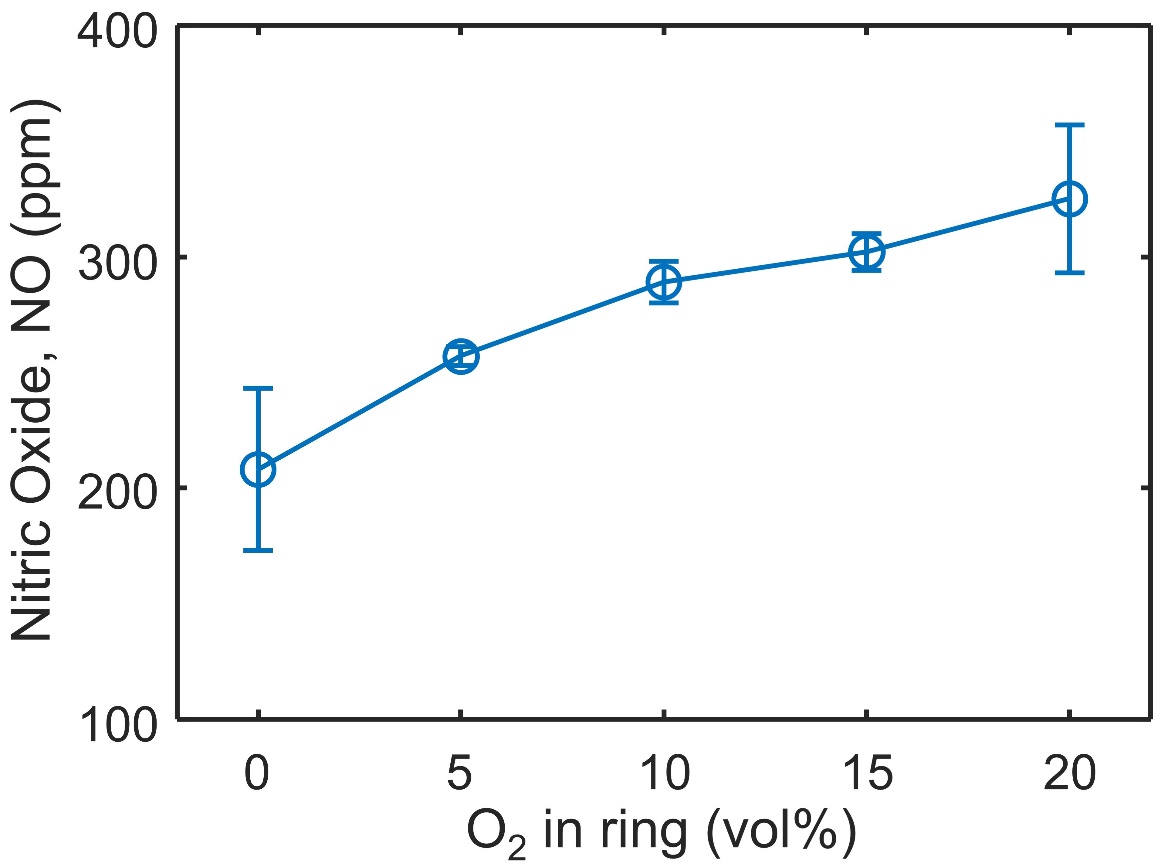


Figure S7: The NO concentration produced at BRD = 30 cm at O_2_ at 0, 5, 10, 15 and 20 vol %.

In addition to the NO emissions produced after air quenching at BRD = 10 – 50 cm (Figure 5), the NO produced during pure N_2_ quenching was also measured as shown in Figure S9.


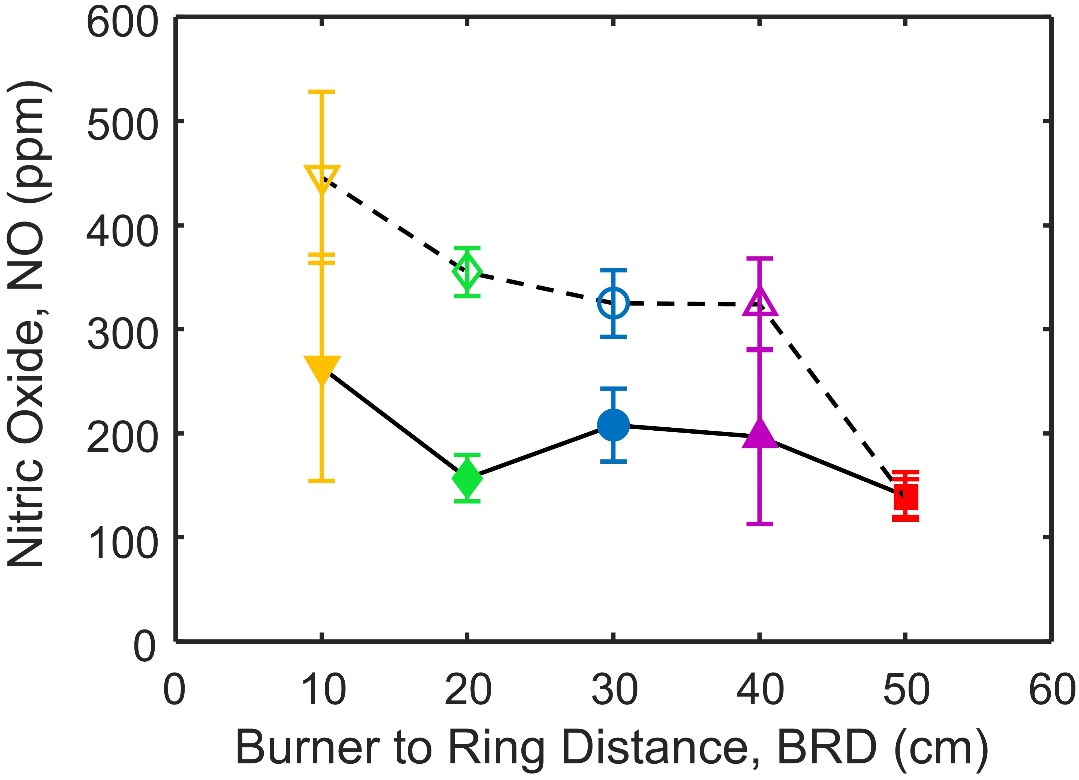


Figure S8: The NO concentration produced with air quenching (open symbols) and pure N_2_ quenching (filled symbols).

The NO concentration emitted from ESC of jet fuel increased linearly with the temperature 5 cm after air injection through the torus ring (Figure 6 and Figure S10, open symbols). This linear relationship (Eq. 1) also held true when pure N_2_ was injected into the system (filled symbols) as depicted in Figure S10 (dot-dashed line).


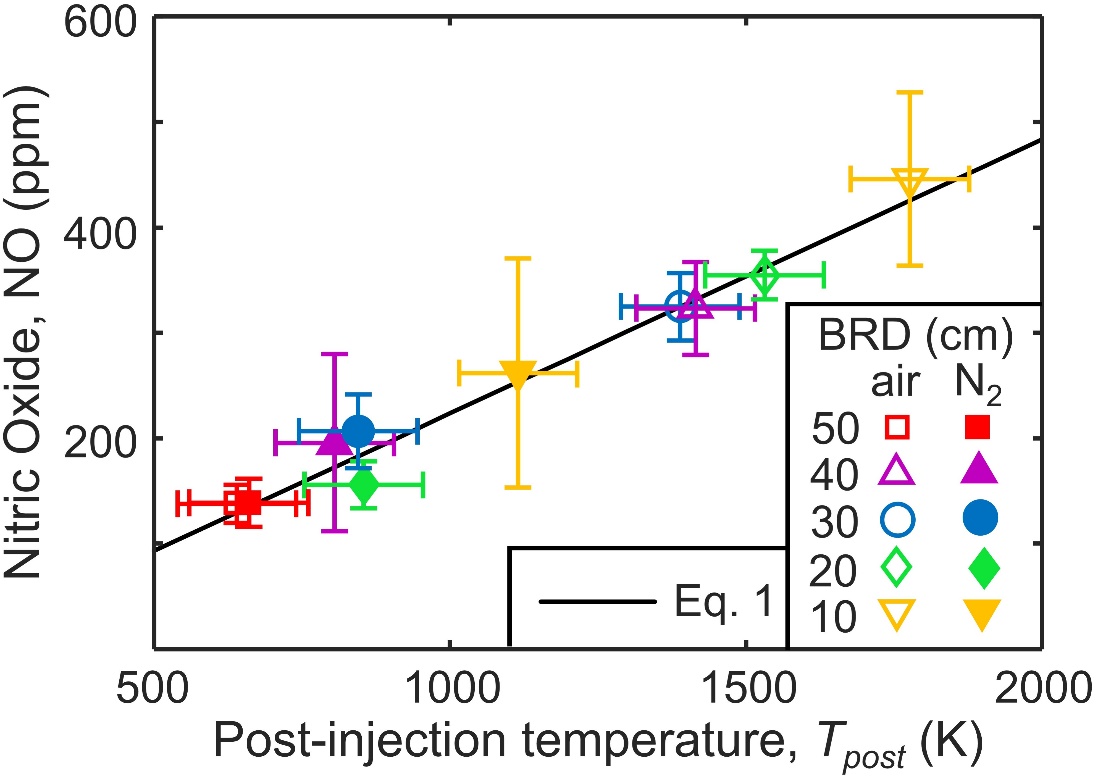


Figure S9: The correlation between post-injection temperature, T_post_, and NO emissions with air (open symbols) and N_2_ quenching (filled symbols) with the best fit (solid line).

Small soot particles are suseptible to diffusion losses in the sampling line leading to a bias in the final measured particle size and therefore, corrections for these losses are recommended when measuring directly behind aircraft engines due to the long (~ 30 – 35 m) sampling lines used [S1]. Here, the sampling lines are shorter, ~ 5.5 m, but the flow rates are also lower, 1.5 LPM to the SMPS. The penetration of particles through a line can be calculated as [S2]:

$$P=\left\{ \begin{aligned} 1-5.5\mu^{\frac{2}{3}}+3.77\mu, &\mu<0.009 \\ 0.819exp\left( -11.5\mu\right)+0.0975exp\left( -70.1\mu\right)+0.0325exp\left( -179\mu\right), &\mu\geq0.009 \end{aligned} \right.$$

where P is the fraction of particles that penetrate through the line and µ is:

$$\mu=\frac{d_{m}L}{Q}$$

where *d*_m_ is the particle mobility diameter, *L* is the length of the line and *Q* is the volumetric flow rate. Here, the penetration of particles as a function of their *d*_m_ is shown in Figure S10. In these circumstances, particle losses are less than 10% for particles larger than 20 nm. By 7 nm, the smallest particle size measured by the SMPS set-up used here, approximately 37% of the particles are lost.


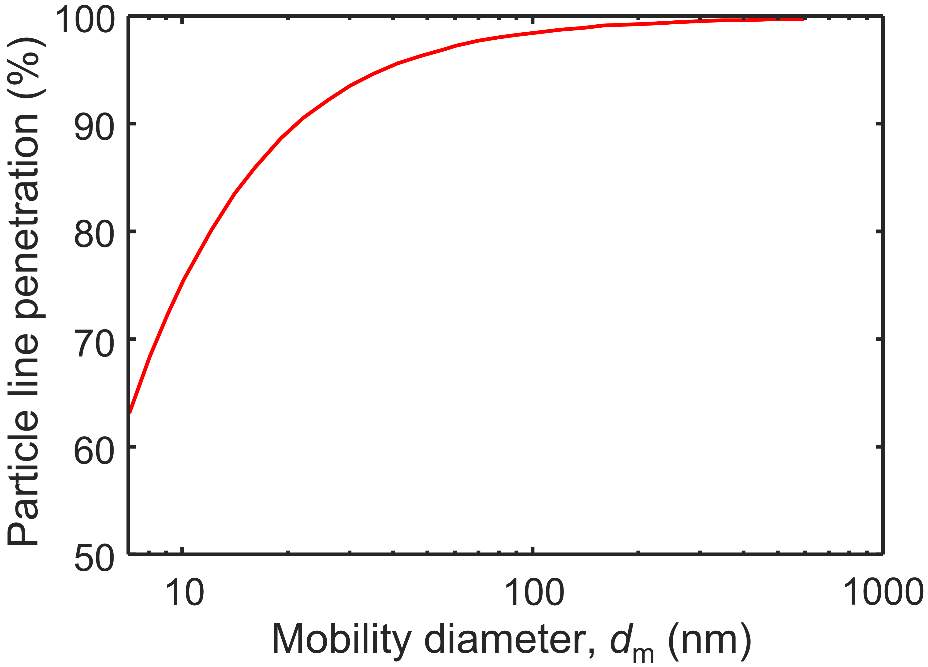


Figure S10: The particle penetration due to diffusional losses for the particles sampled by the SMPS with a flow rate of 1.5 LPM and a 5.5 m sampling line.

**References:**

[S1] E. Durand, L. Durdina, G. Smallwood, M. Johnson, C. Spirig, J. Edebeli, M. Roth, B. Brem, Y. Sevcenco, A. Crayford, Correction for particle loss in a regulatory aviation nvPM emissions system using measured particle size, J Aerosol Sci 169 (2023) 106140. https://doi.org/10.1016/j.jaerosci.2023.106140.

[S2] W.C. Hinds, Aerosol Technology: Properties, Behavior, and Measurement of Air Borne Particles, second ed., John Wiley & Sons, Hoboken, New Jersey, 1999.
